# Rare *BANF1* Alleles and Relatively Frequent *EMD* Alleles Including ‘Healthy Lipid’ Emerin p.D149H in the ExAC Cohort

**DOI:** 10.3389/fcell.2019.00048

**Published:** 2019-04-05

**Authors:** Tejas Dharmaraj, Youchen Guan, Julie Liu, Catherine Badens, Benedicte Gaborit, Katherine L. Wilson

**Affiliations:** ^1^Department of Cell Biology, Johns Hopkins University School of Medicine, Baltimore, MD, United States; ^2^INSERM, MMG, Aix Marseille Université, Marseille, France; ^3^INSERM, INRA, C2VN, Aix Marseille Université, Marseille, France

**Keywords:** barrier to autointegration factor 1, emerin, laminopathy, LDL cholesterol, triglycerides, progeria, mechanotransduction

## Abstract

Emerin (*EMD*) and barrier to autointegration factor 1 (*BANF1*) each bind A-type lamins (*LMNA*) as fundamental components of nuclear lamina structure. Mutations in *LMNA*, *EMD* and *BANF1* are genetically linked to many tissue-specific disorders including Emery-Dreifuss muscular dystrophy and cardiomyopathy (*LMNA*, *EMD*), lipodystrophy, insulin resistance and type 2 diabetes (*LMNA*) and progeria (*LMNA*, *BANF1*). To explore human genetic variation in these genes, we analyzed *EMD* and *BANF1* alleles in the Exome Aggregation Consortium (ExAC) cohort of 60,706 unrelated individuals. We identified 13 rare heterozygous BANF1 missense variants (p.T2S, p.H7Y, p.D9N, p.S22R, p.G25E, p.D55N, p.D57Y, p.L63P, p.N70T, p.K72R, p.R75W, p.R75Q, p.G79R), and one homozygous variant (p.D9H). Several variants are known (p.G25E) or predicted (e.g., p.D9H, p.D9N, p.L63P) to perturb BANF1 and warrant further study. Analysis of *EMD* revealed two previously identified variants associated with adult-onset cardiomyopathy (p.K37del, p.E35K) and one deemed ‘benign’ in an Emery-Dreifuss patient (p.D149H). Interestingly p.D149H was the most frequent emerin variant in ExAC, identified in 58 individuals (overall allele frequency 0.06645%), of whom 55 were East Asian (allele frequency 0.8297%). Furthermore, p.D149H associated with four ‘healthy’ traits: reduced triglycerides (-0.336; *p* = 0.0368), reduced waist circumference (-0.321; *p* = 0.0486), reduced cholesterol (*-*0.572; *p* = 0.000346) and reduced LDL cholesterol (-0.599; *p* = 0.000272). These traits are distinct from *LMNA*-associated metabolic disorders and provide the first insight that emerin influences metabolism. We also identified one novel in-frame deletion (p.F39del) and 62 novel emerin missense variants, many of which were relatively frequent and potentially disruptive including p.N91S and p.S143F (∼0.041% and ∼0.034% of non-Finnish Europeans, respectively), p.G156S (∼0.39% of Africans), p.R204G (∼0.18% of Latinx), p.R207P (∼0.08% of South Asians) and p.R221L (∼0.15% of Latinx). Many novel BANF1 variants are predicted to disrupt dimerization or binding to DNA, histones, emerin or A-type lamins. Many novel emerin variants are predicted to disrupt emerin filament dynamics or binding to BANF1, HDAC3, A-type lamins or other partners. These new human variants provide a foundational resource for future studies to test the molecular mechanisms of BANF1 and emerin function, and to understand the link between emerin variant p.D149H and a ‘healthy’ lipid profile.

## Introduction

Emerin is a conserved ‘LEM-domain’ component of nuclear lamina networks, which have central roles in nuclear structure and function (Simon and Wilson, 2011; Barton et al., 2015; Gruenbaum and Foisner, 2015). Emerin, an integral membrane protein, localizes mainly at the inner membrane of the nuclear envelope (NE) and binds directly to lamins (nuclear intermediate filaments), LINC complexes and an essential chromatin protein named BANF1 (Berk et al., 2013b; Jamin and Wiebe, 2015; Janin et al., 2017). Nuclear lamina proteins have multiple and varied roles from mitosis to tissue-specific signaling (Gerace and Tapia, 2018). For example, during telophase of mitosis, segregating daughter chromosomes are coalesced by BANF1 (Dharmaraj and Wilson, 2017; Samwer et al., 2017). BANF1 then cooperates with emerin, other LEM-domain proteins and lamins to reassemble the nucleus (Liu et al., 2000, 2003; Dechat et al., 2004; Margalit et al., 2005; Barkan et al., 2012; Berk et al., 2013b) and re-establish tissue-specific 3D genome organization (Zullo et al., 2012; Demmerle et al., 2013; Harr et al., 2015; Stevens et al., 2017; van Steensel and Belmont, 2017).

Highlighting these complex roles, missense mutations in lamins and NE membrane proteins cause tissue-specific disorders, termed ‘laminopathies’ (Worman and Schirmer, 2015; Janin et al., 2017). Hundreds of disease-causing variants are reported for the gene encoding A-type lamins (LMNA; Bonne et al., 2004; Dittmer and Misteli, 2011). We previously identified novel *LMNA* missense alleles at frequencies as high as ∼0.4% in specific ethnic groups (Florwick et al., 2017) among 60,706 unrelated individuals (Lek et al., 2016). This raised questions about potential genetic variation in two key lamin-binding proteins: emerin (*EMD*) and BANF1 (*BANF1*).

Emerin is expressed in all tissues and has diverse roles in signaling, cell proliferation, mechanotransduction, transcriptional regulation and 3D genome organization (Demmerle et al., 2013; Le et al., 2016; Iyer et al., 2017; Kirby and Lammerding, 2018). Emerin is specifically important in striated muscle (Barton et al., 2015; Iyer et al., 2017), since loss of emerin causes X-linked recessive EDMD1 (Bonne et al., 2004). EDMD1 is characterized by early contractures of the Achilles, elbow, and neck tendons, progressive muscle weakness and wasting, and dilated cardiomyopathy that can lead to sudden and potentially lethal cardiac arrest. In cardiomyocytes, loss of emerin enhances MAP kinase-dependent gene expression, perturbing heart physiology (Muchir et al., 2007; Huber et al., 2009; Stubenvoll et al., 2015). The same disease, EDMD, is also linked to mutations in proteins that bind emerin including A-type lamins, nesprin-1, nesprin-2, Sun1, Sun2 and LUMA/Tmem43 (Dobrzynska et al., 2016; Pillers and Von Bergen, 2016).

Emerin specifically mediates nuclear responses to mechanical force (Lammerding et al., 2005; Le et al., 2016; Qi et al., 2016; Lele et al., 2018), particularly on stiff substrates (Willer and Carroll, 2017). Emerin is needed to activate mechanoresponsive genes (Lammerding et al., 2005; Le et al., 2016), influences cytoskeletal F-actin networks (Chang et al., 2013) and regulates the SRF-Mkl1 coactivator complex on stiff substrates (Ho et al., 2013; Willer and Carroll, 2017). ‘Pulling’ forces applied to LINC complexes on isolated nuclei cause Tyr-phosphorylation of emerin at unidentified sites, dependent on the integrity of two Tyr residues targeted by Src (emerin Y74 and Y95; Tifft et al., 2009; Guilluy et al., 2014). However, the mechanisms by which emerin, an intrinsically disordered ‘transformer’ protein (Samson et al., 2016), senses force and signals in response to force are open questions. Emerin has at least two modes of self-association with the potential to form networks or filaments (Berk et al., 2014; Herrada et al., 2015; Samson et al., 2017). Other partners include nucleo/cytoskeletal proteins (e.g., F-actin, myosin 1c, tubulin), nuclear membrane proteins (e.g., LAP1, Samp1) and transcriptional regulators such as HDAC3, b-catenin and Lmo7 (Holaska et al., 2006; Berk et al., 2013b; Shin et al., 2013; Barton et al., 2015; Vijayaraghavan et al., 2018).

Additional partners and roles for emerin are emerging in disparate tissues. For example, emerin, BANF1 and lamin A/C associate with the sigma-1 receptor in neurons; association is enhanced by the sigma-1 receptor agonist, cocaine, leading to transcriptional repression of a gene, *MAOB1*, the product of which degrades dopamine (Tsai et al., 2015). The bacterium *Chlamydia psittaci*, which causes rapid and potentially lethal pneumonia, targets emerin and emerin-associated nuclear membrane proteins (Mojica et al., 2015). In models of breast and prostate cancer, loss of emerin correlates with increased metastatic potential (Hu et al., 2011; Wozniak et al., 2013; Reis-Sobreiro et al., 2018). Mechanisms and partners for emerin in these contexts are unexplored.

To our knowledge, only one *BANF1* variant is reported in humans. This variant, p.A12T, was identified in two homozygous individuals and genetically linked to Nestor-Guillermo progeria syndrome (Puente et al., 2011). This variant perturbs BANF1 binding to dsDNA (Paquet et al., 2014) and weakens BANF1 binding to a progeria-related surface on the Ig-fold domain of lamins A and C (Samson et al., 2018). BANF1 is the conserved partner for all LEM-domain proteins (Laguri et al., 2001), and also binds lamins (Holaska et al., 2003; Samson et al., 2018). Indeed, BANF1 can bind emerin and lamin A simultaneously (Bengtsson and Wilson, 2006; Samson et al., 2018). BANF1 is essential during mitosis: BANF1 collects segregating chromosomes within a single nucleus (Samwer et al., 2017; Dharmaraj and Wilson, 2017) and coordinates with lamins and LEM-domain proteins to reassemble nuclear lamina structure (Haraguchi et al., 2008; Simon and Wilson, 2011). BANF1 is mislocalized in cells that express disease mutations in lamin A (Capanni et al., 2012) and is immobilized by fasting (Bar et al., 2014). BANF1 is an epigenetic regulator (Montes de Oca et al., 2011; Oh et al., 2015) and has roles in chromatin organization (Loi et al., 2015), antiviral defense (Kobayashi and Haraguchi, 2015; Wiebe and Jamin, 2016), suppression of endoreplication (Brayson et al., 2018; Wang et al., 2018) and necrotic pyknosis (Hou et al., 2016). BANF1 regulates the insertion of endogenous mobile elements in eukaryotic genomes; this activity is exploited by retroviruses including HIV-1 (Harris and Engelman, 2000; Wang et al., 2014; Jamin and Wiebe, 2015).

DNA sequencing of human populations has the potential to reveal novel phenotypes or missense variants in specific proteins-of-interest, as sources of insight and information for mechanistic studies. This strategy, applied to the gene encoding A-type lamins (*LMNA*), revealed unexpectedly high frequencies of disease-causing *LMNA* alleles in specific ethnic groups, and linked a specific missense variant to type 2 diabetes (Florwick et al., 2017). Other key components of nuclear lamina structure, emerin (*EMD*) and BANF1 (*BANF1*), were unexplored. We therefore analyzed *EMD* and *BANF1* variants in the Exome Aggregation Consortium (ExAC) cohort of 60,706 unrelated individuals (Lek et al., 2016), which includes exome sequences from men and women with diverse ancestries (8.6% African, 9.5% Latinx, 7.1% East Asian, 5.4% Finnish, 55% non-Finish European, 13.6% South Asian, 0.7% other). About half of these individuals either have a specific condition (heart disease, type 2 diabetes, schizophrenia or other disorders) or serve as controls for each condition. We anticipated few loss-of-function *EMD* alleles, since ExAC excludes individuals with severe childhood-onset disorders such as EDMD1. We anticipated few alleles for *BANF1*, given its conservation and essential roles (Zheng et al., 2000; Montes de Oca et al., 2005; Samwer et al., 2017). Highlighting the value of population diversity represented in ExAC, we identified 14 novel *BANF1* missense alleles and many novel *EMD* alleles, ten of which were relatively frequent in specific ethnic populations. We discovered that emerin variant p.D149H, identified in 0.8% of East Asians, associates with a healthy lipid profile including reduced triglycerides and reduced LDL cholesterol. Our detailed structure-function analysis predicts that many identified BANF1 and emerin variants are potentially disruptive and warrant further study.

## Materials and Methods

### ExAC Database Searches

Version 0.3.1 ExAC was queried for ‘*EMD*’ and ‘*BANF1’* via the ExAC Browser (Beta), selecting only for variants that passed the quality assurance filter (Lek et al., 2016; ExAC database, 2018). The data was narrowed to include in-frame deletions and missense, synonymous and splice variants, and exported in the .csv file format. To identify variants associated with broadly defined ‘psychiatric disease’ (schizophrenia, bipolar disorder, Tourette syndrome), we compared two variant call files from ExAC version 0.3. The first file (ExAC.r0.3.sites.vep.vcf) contained all ExAC variants; the second file (ExAC.r0.3.nonpsych.sites.vcf) contained all variants not found in the psychiatric cohort. By subtracting the ‘nonpsych’ variants from the full cohort using the UNIX ‘comm’ tool in a shell script, further selecting for emerin and BANF1 alleles, and manually curating the final list to handle the consequences of minimal and multi-allelic representation, we were able to generate a file of emerin and BANF1 variants unique to the psychiatric cohort. To subset against TCGA, we subtracted the variants not reported in the TCGA dataset (ExAC_nonTCGA.r0.3.1.sites.vep.vcf) from the full cohort to generate a list of potentially ‘cancer-unique’ alleles.

### Laminopathy Database

To search for previously identified variants, we queried the Universal Mutation Database and the Leiden Muscular Dystrophy Pages (powered by Leiden Open Variation Database v2.0; accessed September 2018) for *EMD* variants. After curating to exclude all non-missense mutations and non-canonical spliceforms, only three variants were shared with ExAC.

### Type 2 Diabetes (T2D) Knowledge Portal

Since we were unable to determine the clinical status of variant carriers in ExAC, we searched the open-access Type 2 Diabetes Knowledge Portal for *EMD* variants. ‘Associations across all datasets’ for each ExAC *EMD* variant were obtained using the variant ID or reference SNP ID (rs) as the search term in the T2D portal ^1^. The only protein-coding variant with significant and ‘high-impact’ associations (p.D149H) was identified solely within the 13K Exome Sequence Analysis cohort, which includes samples from 13,007 individuals (half with T2D, half controls) representing five ancestries: European (∼5,000 individuals), African-American, East Asian, South Asian and Hispanic (∼2,000 individuals each). All p.D149H-related statistics come from the analysis reported in the T2D Knowledge Portal.

### Statistical Analysis to Query Potential Sex Distribution Bias

To determine if the sex distribution of variants differed from their expected distribution, we calculated expected distributions based on the ExAC sampling of the sex distribution for each ethnic sub-group in which the allele was most concentrated as a first approximation. For example, the allele producing variant p.N91S was found in 19 individuals of non-Finnish European (nFE) ethnicity, so the proportion of males in the nFE sample of ExAC (56.16%) was used to estimate the expected male allele frequency. To compare the expected versus observed sex distributions, we conducted a chi-squared goodness-of-fit test with 1 degree of freedom. No differences in male/female distribution were found to be significant. Even p.R207P, identified only in men, was explained by a very high proportion of males in the South Asian subgroup where this allele was concentrated.

### Disorder Prediction

To predict intrinsically disordered regions in ExAC variants, variant emerin sequences were submitted as queries to PONDR-FIT VL-XT^2^. Raw VL-XT output scores are real numbers from 0 to 1, where 0 is ‘order’ and 1 is ‘disorder,’ and were plotted.

### Hydrophobicity and Helical Wheel Plots

Kyte-Doolittle hydrophobicity plots^3^ were used to assess the impact of variants located in the C-terminal transmembrane domain. Helical wheel projections showing the positions of variants in the emerin transmembrane domain (residues 226–244) were generated using the Emboss pepwheel tool with default settings^4^. ΔG values for the insertion of transmembrane helices into membranes were calculated using the ΔG prediction server v1.0^5^ (Hessa et al., 2007).

### Structural Depictions

PyMOL version 2.2 was used to generate ray-traced images of the solution NMR structure of the emerin LEM domain (RCSB Protein Data Bank ID: 2ODC) and the crystal structure of BANF1 in complex with the emerin LEM domain (RCSB Protein Data Bank ID: 2ODG).

## Results

ExAC results for *BANF1* are presented first, followed by emerin variants for which we provide allele frequencies, molecular analysis and clinical phenotype.

### BANF1 Variants in ExAC

We were frankly surprised to find 14 *BANF1* missense alleles, one predicted splice acceptor allele and one frameshift allele (p.F59PfsTer50) in ExAC (Table 1 and Supplementary Table S1). The frameshift (p.F59PfsTer50) and splice acceptor alleles are likely to yield unstable polypeptides, and were not considered further. Missense variant p.S22R was identified in three individuals (all African), for an allele frequency of 0.02905% in Africans. All other *BANF1* missense variants were limited to one or two individuals and, surprisingly, one individual was homozygous for the variant identified (p.D9H; Table 1). We also evaluated 3′-UTR alleles of *BANF1* (Supplementary Table S1) because this region is functionally important in cervical cancer, where *BANF1* mRNA is suppressed by miRNA-203 via binding to 3′-UTR nucleotides 138–159 (Mao et al., 2015). This site was not affected by *BANF1* 3′-UTR alleles in ExAC, but might be affected by 3′-UTR alleles reported in the dbSNP database: c.^∗^143T > C (rs1478691828), c.^∗^144C > T (rs1366306879), c.^∗^146C > G (rs535232397), c.^∗^152C > T (rs1442694445). In summary, we identified 14 BANF1 missense variants; all were novel in biology, and most are non-conservative substitutions that might perturb function.

**Table 1:** *BANF1* missense, splice acceptor site and frameshift alleles identified in ExAC.

Variant	Allele count	Comments (ethnic allele frequency)
c.-16-1G > C	1	Splice acceptor site; Finnish (0.01518%)
p.T2S	1	Missense; nF-European (0.001510%)
p.H7Y	2	Missense; 2/2 nF-European (0.003015%)
p.D9N	2	Missense; 2/2 nF-European (0.003013%)
p.D9H	2	Missense; 1 **homozygous** S. Asian (0.01213%)
p.S22R	3	Missense; 3/3 African (0.02905%)
p.G25E	1	Missense; nF-European (0.001503%)
p.D55N	1	Missense; nF-European (0.001498%)
p.D57Y	1	Missense; Latinx (0.008639%)
p.F59PfsTer50	1	Frameshift; Latinx (0.008639%)
p.L63P	1	Missense; African (0.009612%)
p.N70T	1	Missense; South Asian (0.006057%)
p.K72R	1	Missense; nF-European (0.001498%)
p.R75W	1	Missense; Latinx (0.008642%)
p.R75Q	2	Missense; 2/2 nF-European (0.002997%)
p.G79R	1	Missense; nF-European (0.001499%)


**FIGURE 1:**
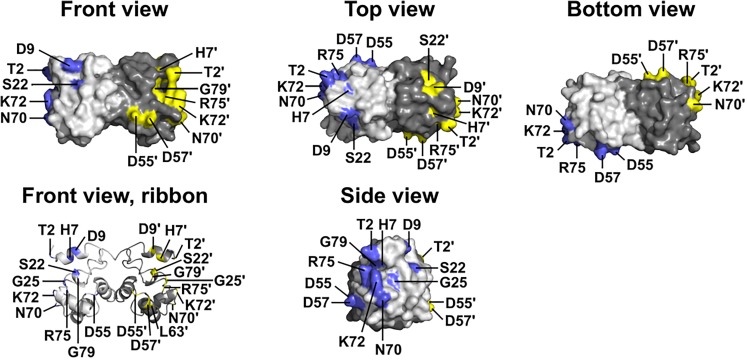
Wildtype BANF1 residues affected by novel ExAC missense variants. Crystal structure (Umland et al., 2000) of the BANF1 dimer showing wildtype residues affected by novel ExAC variants (RCSB Protein Data Bank ID: 1CI4). ‘Top’ and ‘Bottom’ views were obtained by rotating the ‘Front view’ 90° along its long axis out of, and into, the page respectively. ‘Side view’ shows the DNA-binding surface of BAF and was obtained by rotating the Front view 90° to the right. Wildtype residues affected by ExAC variants are colored blue in the pale-gray subunit, and yellow in the dark-gray subunit. Plain and ‘prime’ numbers indicate affected residues in the pale-gray and dark-gray subunits, respectively. Ribbon depiction of the ‘Front view’ reveals residues involved in the dimer interface, and a buried α-helical residue that we predict is disrupted by variant p.L63P.

Two BANF1 missense variants are considered disruptive. Variant p.L63P is likely to disrupt BANF1 folding or dimerization, since WT Leu63 is buried in helix a4 (Figure 1, Ribbon view); whether the resulting protein is degraded or stable is unknown. Interestingly, variant p.G25E was previously created *in vitro* to disrupt BANF1 dimerization: the resulting protein is stable as a monomer, shows no detectable binding to DNA, histones or emerin (Umland et al., 2000; Montes de Oca et al., 2005), and did not disrupt higher-order chromatin structure in *Xenopus* egg extracts (Segura-Totten et al., 2002). When over-expressed in HeLa cells, however, GFP-fused BANF1-G25E interfered with the telophase ‘core’ localization and post-mitotic re-assembly of emerin, LAP2β and lamin A (Haraguchi et al., 2001). Whether p.G25E behaves dominantly or recessively when co-expressed at normal levels with WT BANF1 is unknown.

The other 12 BANF1 missense variants all affect surface-exposed residues (Figure 1), allowing us to evaluate potential molecular impacts. Figure 1 shows ‘front,’ ‘top,’ ‘bottom’ and ‘side’ views of BANF1. WT residues affected by ExAC variants are colored blue in one subunit, yellow in the other subunit (Figure 1). Variants p.D55N and p.D57Y are non-conservative substitutions visible on the ‘front’ and ‘side’ surfaces (Figure 1); the role of the ‘front’ surface is unclear, but may influence binding to histones (Montes de Oca et al., 2005). ExAC variants p.H7Y, p.D9N, p.D9H and p.S22R probably affect the integrity of the ‘top’ surface, but are different from residues (e.g., V11, A12, P14, E83, D86, A87, F88) that contact the Ig-fold of lamins A/C (Samson et al., 2018). Variants p.D9N and p.D9H are potentially more disruptive than an *in vitro* mutation (p.D9A) that reduced binding to DNA, histones and emerin (Umland et al., 2000; Montes de Oca et al., 2005), but retained the ability to hyper-condense chromatin (Segura-Totten et al., 2002). No variants affected residues on the ‘bottom’ surface (Figure 1), which binds the LEM-domain (via BANF1 residues R37, F39, Q48, V51, L52, L58, E61 and W62; Samson et al., 2018). Interestingly most ExAC variants (e.g., p.H7Y, p.D9N, p.D9H, p.S22R, p.D57Y, p.N70T, p.R75W, p.R75Q, p.G79R) are likely to perturb the ‘side’ surface (Figure 1), responsible for binding to DNA (via BANF1 residues K6, G25, G27, V29 and L30, aided by S4, Q5, A71 and R75 (Bradley et al., 2005); two variants (p.R75W, p.R75Q) affected a residue involved in DNA contact. A previously studied *in vitro* substitution, p.R75E, reduced binding to DNA and emerin, but enhanced binding to histones (Montes de Oca et al., 2005). Further studies are needed to determine if any of these novel variants affect BANF1 dimerization, binding to histones, BANF1-dependent epigenetic regulation (Montes de Oca et al., 2009, 2011) or other roles (Jamin and Wiebe, 2015).

### Emerin Variants in ExAC

Overall, *EMD* alleles were considered rare (defined as < 1% of the entire ExAC population), comprising 42 synonymous alleles, six nucleotide changes in splice regions with no suggested consequence, 64 missense alleles and two in-frame deletions (Supplementary Table S2). No *EMD* alleles associated with either cancer or broadly defined psychiatric disease, as determined by subsetting respectively against TCGA and the psychiatric disease cohort in ExAC (see Materials and Methods). Emerin is encoded by an X-linked gene, which allowed us to assess variant distributions in men (hemizygous) versus women (heterozygotes). No *EMD* alleles were homozygous (Supplementary Table S2). A potential sex-distribution bias of two variants, p.G156S and p.R207P, which appeared to be enriched in women and men, respectively, was an artifact of skewed representation of women versus men in specific ethnic groups (see Materials and Methods).

**FIGURE 2:**
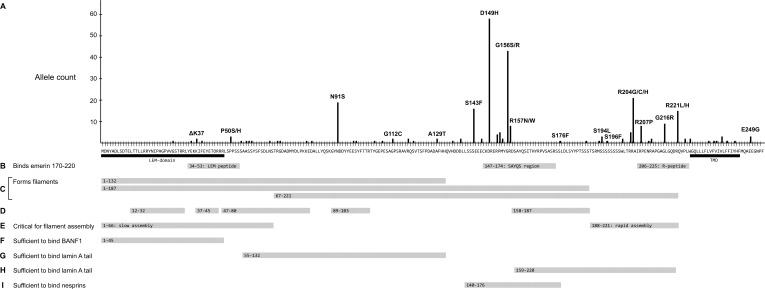
Schematic summary of all identified emerin missense and in-frame deletions in ExAC in relation to functional regions of emerin. **(A)** Amino acid sequence of wildtype emerin. *Y*-axis shows the number of variant alleles at each position (‘Allele count’). Each black dot indicates one affected individual. Some variants are named; for a full list see Supplementary Table S2. Black bars indicate the LEM-domain (Cai et al., 2007) and transmembrane domain (‘TMD’). **(B)** Peptides that mediate intermolecular self-association (Berk et al., 2014). Gray bars indicate three peptides specifically recognized by emerin residues 170–220, named the ‘LEM-peptide’ (residues 34–53), ‘SAYQS region’ (residues 147–174) and ‘R-peptide’ (residues 206–225). **(C-E)** Regions involved in the assembly of curvilinear filaments *in vitro* (Herrada et al., 2015; Samson et al., 2018). **(C)** Gray bars indicate regions that form filaments either slowly (residues 1–132 or 1–187) or rapidly (residues 67–221) *in vitro*. **(D)** Gray bars depict residues that resist digestion with protease, thereby identified as backbone (protected) components of emerin filaments. **(E)** Gray bars depict residues 1–66, critical for (slow) self-assembly of LEM-containing constructs, and residues 188–221, critical for (rapid) self-assembly of residues 67–221. **(F)** Gray bar indicates residues sufficient to bind BANF1 *in vitro*. **(G)** Gray bar indicates residues deduced as sufficient to bind mature lamin A residues 385-646 and, with lower affinity to lamin B1 residues 395–586 (Berk et al., 2014). **(H)** Gray bar indicates a second region of emerin that independently binds mature lamin A residues 385–646 and, with lower affinity to lamin B1 residues 395–586 (Berk et al., 2014). **(I)** Gray bar indicates residues sufficient to bind either nesprin-1 or nesprin-2 *in vitro* (Wheeler et al., 2007). Not depicted: Sun1 and Sun2 bind via deduced emerin residues 134–221 (Haque et al., 2010).

Further analysis focused on *EMD* missense and in-frame deletion alleles (henceforth ‘variants’); 39 variants were unique (identified in a single individual; Supplementary Table S2) and 27 were identified in two or more (up to 58) individuals (Table 2). The positions of all 66 variants with respect to the amino acid sequence of emerin are depicted in Figure 2A, with bar heights indicating the number of affected individuals. Residues known to be involved in self-association, filament formation or binding to BANF1, lamin A or nesprins are also depicted (Figure 2B–I).

**Table 2:** *EMD* missense and in-frame-deletion alleles identified in 2+ individuals in ExAC.

Variant	# Individuals	Allele frequency (%)	Ethnic concentration^∗^
p.K37del	2	0.002529	n/a
p.P50S	2	0.002547	n/a
p.N91S	19	0.02236	19/19 nF-European (0.04123%)
p.G112C	2	0.002333	n/a
p.R118H	2	0.002333	n/a
p.A129T	2	0.002336	n/a
p.D138N	2	0.002294	n/a
p.S143F	16	0.01834	16/16 nF-European (0.03365%)
p.E146K	2	0.002291	n/a
p.D149H	58	0.06645	55/58 East Asian (0.8297%)
p.R152C	3	0.003436	3/3 nF-European (0.0066298%)
p.P153L	5	0.005726	3 ethnicities^∗∗^
p.G156S	34	0.03893	33/34 African (0.3894%)
p.G156R	9	0.01031	9/9 nF-European (0.01889%)
p.R157Q	7	0.008016	5/7 nF-European (0.01049%) 2/7 Latinx (0.02149%)
p.S194L	3	0.003433	3/3 South Asian (0.02964%)
p.W200R	2	0.00229	n/a
p.R203H	4	0.00458	3/4 East Asian (0.04527%)
p.R204G	17	0.01947	17/17 Latinx (0.1826%)
p.R204C	3	0.003436	3/3 nF-European (0.006296%)
p.R207P	8	0.009166	8/8 South Asian (0.07917%)
p.G216R	9	0.01033	9/9 African (0.1064%)
p.R221L	14	0.0161	14/14 Latinx (0.1505%)
p.P224L	2	0.002301	n/a
p.W226S	2	0.002303	n/a
p.V238I	3	0.003477	2/3 nF-European (0.004255%), 1/3 Latinx (0.01078%)
p.E249G	3	0.00361	3/3 nF-European (0.006632%)


*^∗^n/a, not applicable; calculated only for alleles identified in 3 or more individuals. ^∗∗^2 European (0.04423%); 2 nF-European (0.004198%); 1 Other (0.1592%).*

### Three Emerin ExAC Variants (p.K37del, p.R203H, p.D149H) Reported in Laminopathy Database

We anticipated little overlap with the laminopathy database, since severe childhood-onset disorders such as EDMD1 are excluded from ExAC (Lek et al., 2016). Indeed, only three emerin ExAC variants were previously reported in the laminopathy database: p.K37del, p.R203H and p.D149H. Variant p.K37del causes the loss-of-function EDMD1 phenotype even though the resulting protein is expressed at normal or near-normal levels (Ellis et al., 1998). Loss of residue K37 destabilizes the LEM-domain (Samson et al., 2017), reduces affinity for BANF1 (Essawy et al., personal communication) and accelerates the rate of emerin self-association as filaments *in vitro* (Samson et al., 2017). Finding two individuals with p.K37del in ExAC was plausible because this variant is known to cause isolated cardiac disease in adult men (hemizygous) and women (heterozygous; Ben Yaou et al., 2007), and cardiac disease is well-represented in ExAC (Lek et al., 2016). A similar case can be made for variant p.R203H (identified in four ExAC individuals), because the only reported EDMD1 patient with this variant showed cardiac symptoms as an adult (39 years; Funakoshi et al., 1999). These results were encouraging in their suggestion that ExAC populations might also include novel variants that specifically affect the heart.

The third ‘known’ variant, p.D149H, was reported in one EDMD1 patient but nevertheless deemed benign with respect to laminopathy. This was exciting because p.D149H was the most-frequent *EMD* variant in ExAC and associated with a novel metabolic phenotype, as described below.

### Ethnic Concentrations of the Most Frequent Emerin Variants in ExAC

The most frequent emerin variants in ExAC, including nine novel variants, each concentrated in a specific ethnic group (Table 2 and Supplemental Table S2). Topping the list was variant p.D149H, identified predominantly in East Asians (55 of 58 individuals) with an allele frequency of 0.8297% in East Asians; the other individuals were South Asian (one) and Latino (henceforth ‘Latinx’; two). Non-Finnish Europeans (henceforth, ‘Europeans’), the largest ethnic population in ExAC, also had the greatest overall number of emerin variants (Table 2). Three variants were identified only in Europeans: p.N91S (all 19 individuals; allele frequency 0.04123%), p.S143F (all 16 individuals; allele frequency 0.03365%) and p.G156R (all 9 individuals; allele frequency 0.01889%), corresponding to frequencies of 1.8 to 4.1 per 10,000 individuals. Variant p.R157Q was identified predominantly in Europeans (5 of 7 individuals; allele frequency 0.01049%); the other two were Latinx. Variant p.G216R was identified only in Africans (all 9 individuals; allele frequency 0.1064%). Variant p.G156S was identified predominantly in Africans (33 of 34 individuals; allele frequency 0.3894%); the other individual was European. Two variants were identified only in Latinx: p.R204G (all 17 individuals; allele frequency 0.1826%) and p.R221L (all 14 individuals; allele frequency 0.1505%). Variant p.R207P was identified only in South Asians (all 8 individuals; allele frequency 0.07917%). In other words, six variants (p.D149H, p.G156S, p.R204G, p.R207P, p.G216R, p.R221L) were identified at frequencies of 10–83 per 10,000 individuals in specific populations. Note that these frequencies may be skewed by multiple factors including distribution artifacts and potential association with disorders (e.g., cardiomyopathy or type 2 diabetes) that are enriched in ExAC.

### Variant p.D149H, Identified in 0.8297% of East Asians, Associates With a ‘Healthy’ Lipid Profile

Variant p.D149H was identified 58 times in ExAC with allele frequencies of 0.0665% overall and 0.8297% in East Asians (Table 2 and Supplementary Table S2). For most disorders, ExAC does not link individuals to clinical phenotypes. However, ExAC includes type 2 diabetes cohorts for whom clinical data is accessible via the T2D Knowledge Portal^6^. To determine if any variants associated with a metabolic phenotype(s), we searched within 100 kb of *EMD* (see Materials and Methods). We found no significant associations with body-mass index, diastolic blood pressure, fasting glucose, fasting insulin, glycated hemoglobin (HbA1c), HDL cholesterol, height, hip circumference, systolic blood pressure, type 2 diabetes or waist-hip ratio. By contrast, four traits showed a significant association with emerin variant p.D149H: reduced triglycerides (effect was -0.336; *p* = 0.0368), reduced waist circumference (effect was -0.321; *p* = 0.0486), reduced cholesterol (effect was -0.572; *p* = 0.000346) and reduced LDL cholesterol (effect was -0.599; *p* = 0.000272). We concluded that emerin variant p.D149H specifically influences metabolism (see Discussion).

### ‘Top-Ten’ Emerin Variants in Regions That Self-Associate and Bind Lamin A and Nesprins

Variant p.N91S is a disruptive mutation in a core (protease-protected) region of emerin filaments (Figure 2D) that also binds lamin A (Figure 2G). Other ‘top-ten’ variants clustered in two regions: variants p.S143F through p.R157Q affect residues in the ‘SAYQS’ region, while variants p.R203H through p.R221L affect residues in the ‘R-peptide’ region, near the transmembrane domain (TMD; Figure 2B). Both regions mediate emerin self-association, as shown in peptide binding studies (Figure 2B; Berk et al., 2014) and biophysical studies of emerin filaments (Figure 2C,D; Herrada et al., 2015; Samson et al., 2017). Both regions also directly bind lamin A (Figure 2H), nesprins (Figure 2I) and other partners (Berk et al., 2014). The substitutions created by variants p.D149H, p.P153L, p.R204G and p.R207P are predicted to perturb one or more of these functions (see Discussion).

### LEM-Domain Variants: One Known (p.K37del) and Three Novel (p.G28A, p.E35K, p.F39del)

**FIGURE 3:**
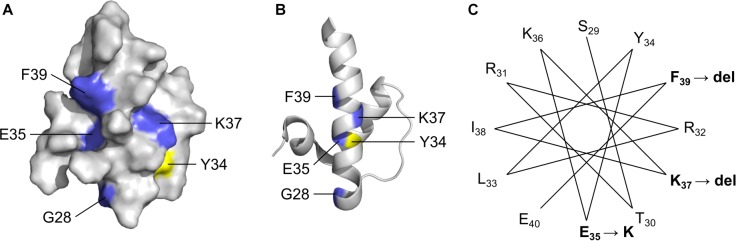
Solution NMR structure (Cai et al., 2007) of the ‘LEM’ domain showing residues affected by ExAC variants (RCSB Protein Data Bank ID: 2ODC). Surface depiction **(A)** and ribbon depiction **(B)** of the wildtype LEM domain. Residues affected by ExAC variants p.G28A, p.E35K, p.K37del and p.K39del are shown in blue. Also depicted in yellow for reference is Tyr34, which directly contacts BANF1 (Cai et al., 2007; Samson et al., 2018). **(C)** A helical projection suggests the longest α-helix, known to be destabilized by p.K37del, is also perturbed by novel variants p.E35K and p.K39del.

**FIGURE 4:**
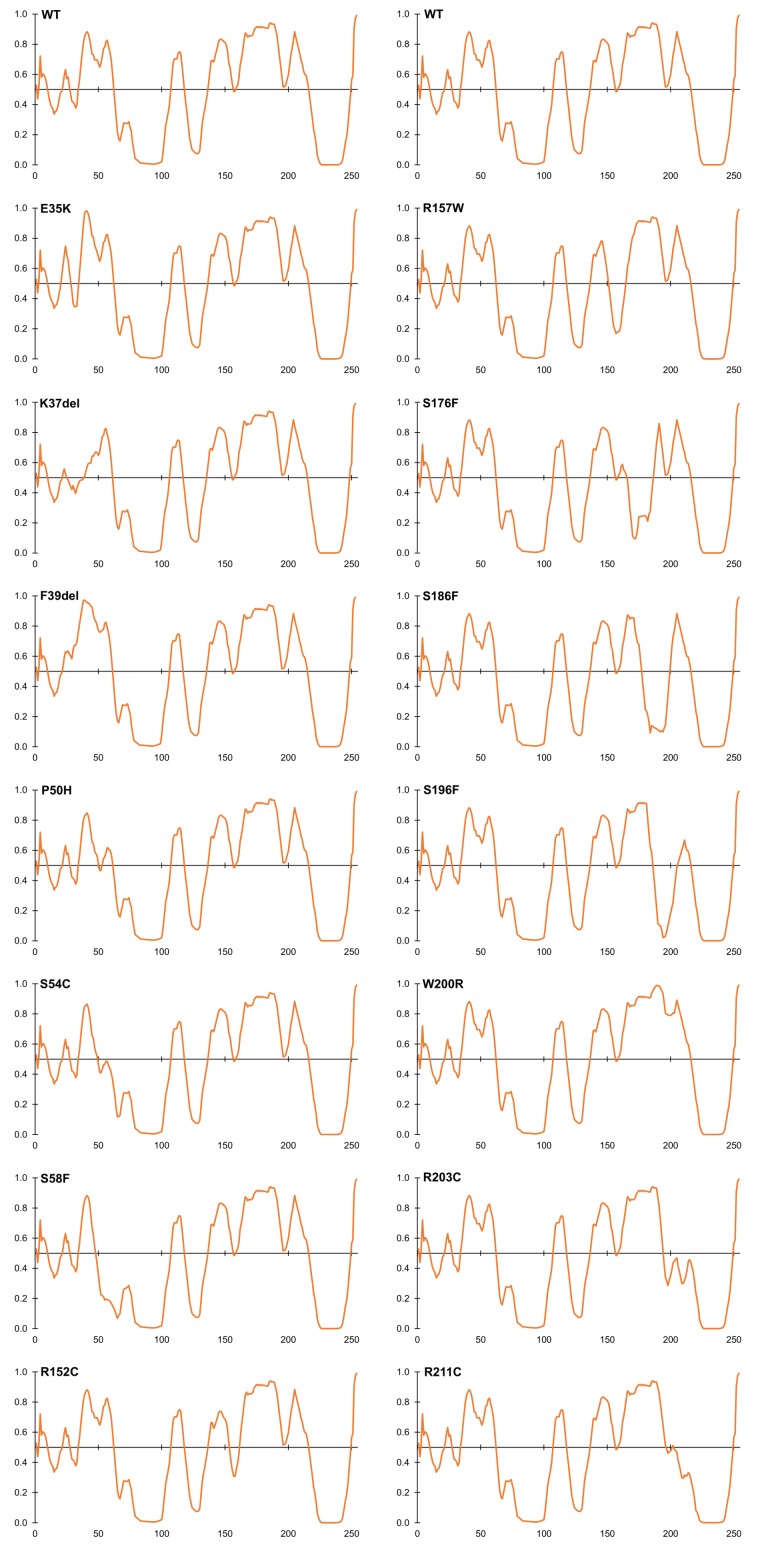
Intrinsic disorder predictions for selected ExAC variants, relative to wildtype emerin (WT). Disorder predictions from PONDR-FIT VL-XT are graphed on the *Y*-axis: disorder increases above the horizontal line and decreases below the line. The *X*-axis indicates amino acid positions in emerin.

Four LEM-domain variants were identified in ExAC: the cardiomyopathy-associated variant p.K37del (discussed above) and three unique novel variants: p.G28A, p.E35K and p.K39del (Supplementary Table S2). WT LEM-domain residues affected by ExAC variants are depicted via surface view (Figure 3A) and ribbon diagram (Figure 3B; PDB code 2ODC; Cai et al., 2007). All four WT residues are surface-exposed (Figure 3A) on the large α-helix (Figure 3B). Residues K35, K37, and F39 each stabilize the α-helix (Figure 3C). Deletion of residue K37 destabilizes the LEM-domain (Samson et al., 2017); we therefore expect the same or worse for variant p.F39del, which is predicted to enhance disorder more than p.K37del (Figure 4). Missense variant p.E35K, which replaces a negative charge (Glu) with a positive charge (Lys), is predicted to increase disorder for residues 21–29 and 35–49 (Figure 4) and thus has the potential to destabilize the LEM-domain. Variant p.G28A is positioned to alter the ‘left’ surface of the LEM-domain (as viewed in Figure 3A). A more-disruptive substitution, p.G28R, is reported for three individuals in the dbSNP database (Praxis fuer Humangenetik Teubingen; ID: rs1064797380). The functions of this surface are unclear, since BANF1 binds the other (‘right’) side (as viewed in Figure 3A) via direct contact with LEM-domain residues Y34 (colored yellow; Figure 3A,B), T13, G24, P25, V27, S29, T30, L33, K36, and K37 (Cai et al., 2007; Samson et al., 2018). Intriguingly, this ‘mystery’ surface of the LEM-domain includes α-helical residues Y34 through R47, which together with disordered residues L48-S53 comprise the ‘LEM-peptide,’ which mediates emerin self-association (Figure 2B) and is largely protease-resistant in the context of emerin filaments (Figure 2D; Samson et al., 2017). Thus, novel ExAC variants p.G28A and p.E35K, along with p.G28R, are predicted to disrupt a conformational change(s) of the LEM-domain that contributes to emerin filament formation, and might also reduce emerin affinity for lamin A, which binds preferentially to emerin filaments (Samson et al., 2018).

### Variants That Perturb Intrinsic Disorder, a Fundamental Property of Emerin

Beyond the LEM-domain, emerin is dominated by intrinsic disorder, as shown by biophysical analysis of emerin residues 50–132 and residues 67–170 (Samson et al., 2016). Disorder is an important quality that allows such ‘transformer’ proteins to bind specific partners with high affinity, and to undergo regulated conformational changes that create new ‘platforms’ for different sets of partners (Uversky, 2011; Wright and Dyson, 2015; Ruan et al., 2018). About 30% of emerin variants in ExAC were predicted by PONDR VL-XT to increase or decrease disorder (Figure 4). Variants p.P50H, p.S54C, p.S57F, and p.S58F are all predicted to reduce or eliminate disorder near the LEM-domain (Figure 4); interestingly, their proximity to known phosphorylation and *O*-GlcNAcylation sites (e.g., S55, S56, S60, S62) suggests they might also interfere with posttranslational control of this proposed ‘hinge’ region (Roberts et al., 2006; Berk et al., 2013a,b). Variant p.S54C, identified in one individual in ExAC, is a novel substitution at residue Ser54. A different substitution, p.S54F, disrupts binding to HDAC3 (Demmerle et al., 2012) and other partners (Berk et al., 2013b) and is sufficient to cause EDMD1 (Ellis et al., 1998).

Several novel variants were also predicted to either reduce (p.R152C, p.R152H, p.P153L) or eliminate disorder (p.R157W) in the conserved ‘SAYQS’ region (Figure 4), which mediates emerin self-association (Figure 2B–D) and binding to nesprins (Figure 2I). Seven novel variants (p.S176F, p.S186F, p.S192F, p.S196F, p.R203C, p.R204C, p.R211C) are predicted to eliminate disorder in the distal region (Figure 4) that is both sufficient for binding to lamin A (Figure 2H) and critical for emerin self-association (Figure 2B–E). Most such variants were identified in only one individual each, except p.R152C and p.R204C (three individuals each) and p.P153L (five individuals; Supplementary Table S2). These variants warrant further study (see Discussion).

### Emerin Variants in Regions Needed for TRC40-Dependent Membrane Insertion

**FIGURE 5:**
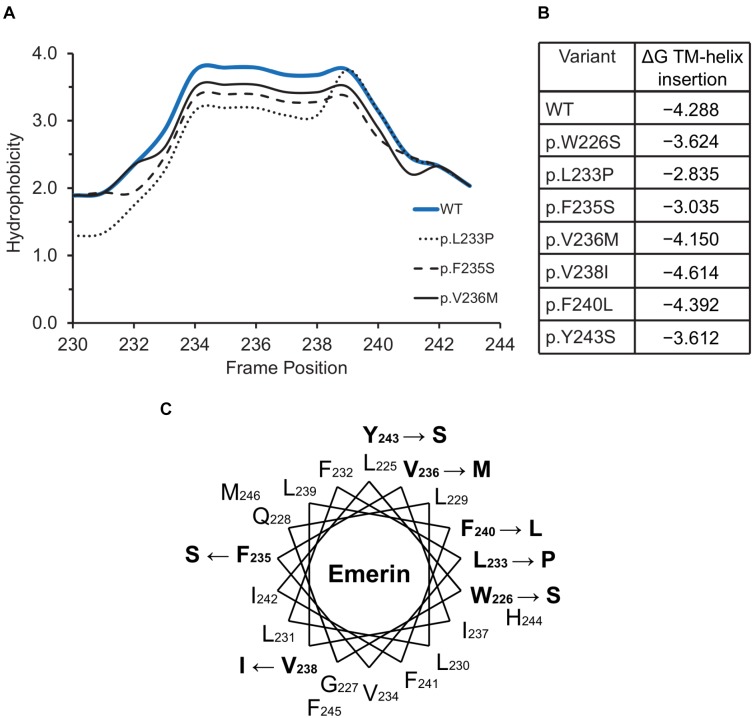
Variants located in or near the emerin C-terminus. **(A)** Kyte-Doolittle hydrophobicity plots of the transmembrane domains of wildtype (WT) emerin and selected ExAC variants. **(B)** Predicted ΔG for transmembrane helix insertion of WT emerin and each indicated ExAC variant. **(C)** Helical wheel projection of the transmembrane domain of emerin (residues 226–244), plotted according to Pfaff and colleagues (Pfaff et al., 2016). WT residues affected by ExAC variants are bold, with an arrow pointing to the substitution.

The hydrophobic C-terminal domain of emerin is recognized by the Transmembrane Recognition Complex 40 (TRC40) pathway for posttranslational insertion into ER membrane (Pfaff et al., 2016). Emerin then diffuses to the NE inner membrane and is retained by binding to A-type lamins (Östlund et al., 2006) and nesprins (Wheeler et al., 2007; Zhang et al., 2007). Several variants including p.L233P, p.F235S and p.Y243S had slightly reduced Kyte-Doolittle hydrophobicity relative to WT emerin (Figure 5A), and less favorable (but still negative) ΔG values for transmembrane helix insertion (Figure 5B). Helical wheel projections of transmembrane residues 226–244 (Figure 5C) showed that variants p.W226S, p.V236M and p.F240L affect conserved residues (Pfaff et al., 2016). Other substitutions (p.L233P, p.F235S, p.Y243S) are likely to disrupt features needed for TRC40-dependent insertion (Figure 5C). These predictions warrant future testing, since most current knowledge about emerin insertion into membranes is based on EDMD-associated frameshifts or deletions that truncate or remove the transmembrane domain (Pfaff et al., 2016). TRC40-dependent insertion is also significantly (∼44%) reduced by EDMD-causing variants p.P183T and p.P183H, located ∼40 residues upstream of the transmembrane domain, through unknown mechanisms (Pfaff et al., 2016). ExAC variants in this upstream region were rare (four unique variants between residues 158–193; Supplementary Table S2). Specific TRC40-dependent targeting to the ER/NE (rather than mitochondria or peroxisomes) requires negatively charged residues on the lumenal C-terminus (Costello et al., 2017; Figueiredo Costa et al., 2018). Emerin has two such residues, one of which is neutralized by variant p.E249G, identified in three individuals in ExAC (Figure 2). We speculate that emerin p.E249G might be inefficiently targeted to the ER/NE, and end up in the wrong organelle.

## Discussion

Population diversity in ExAC was key to discovering many novel variants in the genes encoding emerin and BANF1. The first thing to keep in mind is that most or all of the *EMD* and *BANF1* alleles in ExAC could be phenotypically silent, a common theme in human genetics. However as highlighted in this report, certain variants have the potential to perturb emerin or BANF1 at the molecular level. Because *EMD* is X-linked, men will express the variant emerin exclusively, increasing the possibility of a clinical phenotype. Women express *EMD* variants exclusively in about 50% of cells, increasing their genetic risk if the variant perturbs emerin function in cardiomyocytes. Novel human variants identified in this study provide new tools to study their molecular roles and potential association with adult-onset heart disease or new physiological roles, as reported here for ‘healthy lipid’ emerin variant p.D149H. We discuss our results for BANF1 first.

### Human BANF1 Variants: Novel and Rare

BANF1 was first discovered as a protein essential for retroviral integration into the genome (Lee and Craigie, 1998). Its atomic structure (obligate dimer) and binding sites for dsDNA (two) and the LEM-domain are solved (Bradley et al., 2005; Cai et al., 2007). Residues involved in binding to dsDNA, histone H3, histone H1 or emerin were characterized by *in vitro* mutagenesis (Umland et al., 2000; Segura-Totten et al., 2002; Montes de Oca et al., 2005). Consistent with its essential roles in mitosis, we found that genetic variation in *BANF1* was constrained. *BANF1* had a loss-of-function intolerance (pLI) score of 0.74, comparable to that of histone H3 (*H3F3A*; pLI score 0.69), and more intolerant than *LMNA* (pLI score 0.99; Florwick et al., 2017).

**FIGURE 6:**
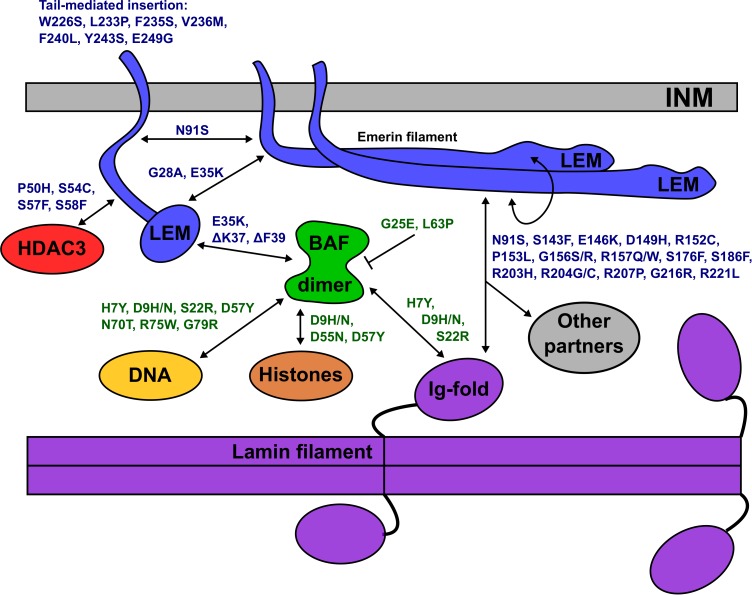
Schematic depiction of the inner nuclear membrane (INM) showing emerin, BANF1, an A-type lamin filament and selected partners (HDAC3, DNA, histones). Emerin polypeptides and variants are shown in blue. BANF1 dimer and variants are shown in green. Lamin filament with four Ig-fold ‘tail’ domains is shown in purple. Double-headed arrows indicate direct association, and each named variant is predicted (or in certain cases, known) to perturb the interaction. Emerin has several modes of self-association and can form filaments (see text). Variant p.G25E inhibits BANF1 dimerization (indicated by the bar) resulting in loss of binding to dsDNA, histones and the LEM-domain; the same is predicted for novel variant p.L63P. ‘Other’ partners not depicted include F-actin, nesprins, β-catenin and other transcription factors (see text). Testing these predictions in future, especially for p.D149H and other ‘high-frequency’ emerin variants, may provide molecular insights into nuclear lamina functions and human disease.

All 14 identified BANF1 missense variants were novel, and most were non-conservative substitutions with the potential to perturb BANF1 structure or function. The known or predicted molecular impacts of BANF1 variants identified in this study are summarized schematically in Figure 6. Further studies are needed to determine if heterozygous BANF1 variants are recessive or dominant. The only previously reported human BANF variant, p.A12T, causes Nestor-Guillermo progeria syndrome when homozygous (Puente et al., 2011). When expressed ectopically in U2OS cells, variant p.A12T caused a significant increase in the percentage of cells with a misshapen nucleus (Paquet et al., 2014). We therefore speculate that heterozygous variants (e.g., p.G25E, p.L63P, p.D9N) and homozygous variant p.D9H might cause cellular phenotypes. The discovery of rare non-conservative human BANF1 variants is exciting and warrants further study in the contexts of nuclear lamina function, cell cycle control, epigenetics, embryonic stem cells, mechano-transduction or novel roles (Haraguchi et al., 2007; Cox et al., 2011; Graham and Burridge, 2016; Brayson et al., 2018; Wang et al., 2018).

### ExAC Variants in or Near the Emerin LEM-Domain

One intriguing question is whether the LEM-domain unfolds or changes conformation *in vivo*. The rate of emerin filament formation *in vitro* is accelerated by mutations that destabilize the LEM-domain (Samson et al., 2017), and further accelerated in the absence of the LEM-domain (Figure 2C,E; Samson et al., 2018; Essawy et al., personal communication). As noted earlier, several ExAC variants (e.g., p.P50H, p.S54C, p.S58F) near the LEM-domain have the potential to disrupt a proposed ‘hinge’ region (Figure 2B–D), either by making it inflexible (Figure 4) or by interfering with cellular control (e.g., phosphorylation vs. *O*-GlcNAcylation at Ser54; Berk et al., 2013a).

### Novel Variants-of-Interest in Lamin-Binding Regions

The molecular basis for lamin A binding to emerin is beginning to emerge. Lamin A binds emerin both directly via emerin residues 55–132 (Figure 2G) or 159–220 (Figure 2H), and indirectly through BANF1 (Samson et al., 2018). Furthermore, the Ig-fold domain of lamin A associates with emerin filaments, not emerin monomers (Samson et al., 2018; Essawy et al., personal communication). Thus, we predict that contact with lamin A is perturbed by non-conservative substitutions in peptides required for emerin self-association (e.g., p.P50S, p.P50H, p.D149H, p.G156S, p.R207P, p.G216R, p.R221L), and by variants in regions that bind lamins (e.g., p.N91S, p.R204G/C/H), as illustrated schematically in Figure 6. For example, emerin variants p.R207P and p.G216R are highly disruptive substitutions in the conserved ‘R-peptide’, which is critical for emerin self-association and binding to lamins (Figure 2B,E,H). Several variants (e.g., p.S143F, p.D149H, p.G156R) have the potential to affect binding to nesprins (Figure 2I; ‘Other partners’ in Figure 6).

### Variants Near Sites of Force-Stimulated Emerin Tyr-Phosphorylation

‘Pulling’ on LINC complexes of isolated nuclei stiffens the nucleus within seconds (Guilluy et al., 2014). This mechanical response requires Src phosphorylation of emerin, and is significantly reduced by substitutions that block phosphorylation at two sites in emerin: Y74F and Y95F (Tifft et al., 2009; Guilluy et al., 2014). The molecular mechanisms of emerin-dependent stiffening are unknown, and might plausibly involve force-induced and/or phosphorylation-induced changes in emerin conformation, self-association and/or binding to lamin A, BANF1 (chromatin), F-actin or myosin 1c (Holaska and Wilson, 2007; Virtanen and Vartiainen, 2017; Lambert, 2018). Testing these models will be an intriguing challenge, since emerin is flexible and self-association is required to bind many other partners including A-type lamins (Figure 6). We speculate that variants such as p.N91S and p.Y105C might perturb Src-dependent phosphorylation at Y74 and Y95 (Table 2; Tifft et al., 2009). Novel ExAC variants also provide a rich source of human variants to test for potential defects in downstream emerin-dependent transcription regulation (Ho et al., 2013; Willer and Carroll, 2017; Kirby and Lammerding, 2018).

### Emerin Variant p.D149H Associated With a ‘Healthy’ (Fasting) Lipid Phenotype

Emerin variant p.D149H, reported as benign with respect to EDMD1, associated significantly with four traits in the Type 2 Diabetes Knowledge Portal: reduced triglycerides and reduced waist circumference, as well as high-impact reductions in LDL cholesterol and cholesterol. To our knowledge, this is the first evidence that emerin directly influences metabolism. The emerin p.D149H phenotype is opposite to that seen in *LMNA*-associated metabolic disease, especially for triglycerides and waist circumference, both of which are elevated in *LMNA*-associated lipodystrophy, insulin resistance and metabolic syndrome (Decaudain et al., 2007; Vadrot et al., 2015; Dutour et al., 2011; Brayson and Shanahan, 2017). In contrast to our previous ExAC study, which linked lamin A variant p.G602S with type 2 diabetes (Florwick et al., 2017), we found no association of any emerin variant with type 2 diabetes.

The traits associated with emerin p.D149H, especially the reduction in LDL (‘bad’) cholesterol, are considered healthy. The magnitude of LDL reduction associated with p.D149H is comparable to the expected effects of drugs such as statins, which aim to reduce LDL cholesterol by ≥50% to decrease risk of adverse cardiovascular events (Ridker et al., 2016). The triglyceride phenotype of emerin p.D149H is similar to that caused by loss-of-function mutations in *ANGPTL4* (angiopoietin-like protein 4), a secreted cytokine that helps maintain low serum triglyceride levels by inhibiting the enzyme, lipoprotein lipase (Dijk and Kersten, 2014). In mice, *ANGPTL4* is expressed mainly in adipose tissue and is upregulated by fasting in both white adipose tissue and liver (Dijk et al., 2018). A missense mutation in *ANGPTL4* (p.E40K), detected in ∼3% of European Americans, associates with reduced triglycerides and increased HDL (‘good’) cholesterol (Romeo et al., 2007, 2009), in proportions comparable to the effect of emerin p.D149H. The p.E40K mutation in *ANGPTL4* also associates with reduced risk of coronary disease (Stitziel and Myocardial Infarction Genetics and CARDIoGRAM Exome Consortia Investigators, 2016).

The emerin p.D149H lipid phenotype raises important questions. What do these ‘healthy’ lipid traits imply about the normal roles of emerin in striated muscle (Brull et al., 2018), or other tissues? Is this emerin phenotype related to the immobilization of BANF1 at the nuclear lamina in response to fasting in *Caenorhabditis elegans* intestinal cells (Bar et al., 2014)? Is this ‘healthy lipid’ phenotype seen in both men and women, or is it less pronounced in women, who express WT emerin in ∼50% of cells? We do not know which tissues are affected by emerin p.D149H, although liver and adipose are plausible candidates. Another open question is whether the p.D149H phenotype is related to, or independent of, the metabolic phenotypes caused by dominant *LMNA* variants. Future exploration of variant p.D149H may provide insight into the protective roles of WT emerin in the heart.

### Where to Go From Here

Exome sequence data from human populations is an emerging resource in biology, with the potential to advance personalized medicine for individuals with well-characterized mutations. Our analysis of *EMD* revealed an unexpected association between emerin variant p.D149H and a ‘healthy lipid’ profile, and showed that nearly 1% (∼0.83%) of East Asians in ExAC carry this variant. Our previous study linked *LMNA* variant p.G602S to type 2 diabetes and showed that ∼0.3% of African Americans carry this allele, and revealed a dominant lipodystrophy-causing variant (p.I299V) in 0.347% of Latinx individuals in ExAC (Florwick et al., 2017). However, many fundamental aspects of nuclear biology are unexplored or incompletely understood, including the molecular connections between emerin, BANF1 and lamin filaments at the NE. Our analysis of three nuclear lamina genes – *LMNA* (Florwick et al., 2017), *EMD* and *BANF1* (this study) – in 60,706 unrelated individuals in ExAC has yielded an unexpected trove of novel variants with the potential to perturb specific interactions at the NE, as shown schematically in Figure 6. These variants and predictions await testing.

## Author Contributions

KW contributed to the conception and first draft. TD, JL, and YG prepared the figures and tables. TD, CB, BG, and KW analyzed the data. All authors contributed to the intellectual input, manuscript preparation and editing.

## Conflict of Interest Statement

The authors declare that the research was conducted in the absence of any commercial or financial relationships that could be construed as a potential conflict of interest. The handling Editor and reviewer KR declared their involvement as co-editors in the Research Topic, and confirm the absence of any other collaboration.

## Footnotes

1 http://www.type2diabetesgenetics.org/variantInfo/variantInfo/X_153609158_G_C

2 http://www.pondr.com

3 https://web.expasy.org/protscale/

4 http://emboss.bioinformatics.nl/cgi-bin/emboss/pepwheel

5 http://dgpred.cbr.su.se/index.php?p=TMpred

6 http://www.type2diabetesgenetics.org/

## Supplementary Material

TABLE S1 List of *BANF1* variant alleles in ExAC.

TABLE S2 List of *EMD* variant alleles in ExAC.

## Abbreviations

*BANF1*: Barrier to autointegration factor 1
*EDMD*: Emery-Dreifuss muscular dystrophy
*LEM-domain*: LAP2-Emerin-MAN1 domain
*LINC*: linkers of the nucleoskeleton and cytoskeleton
*T2D*: type 2 diabetes
*TCGA*: The Cancer Genome Atlas. *WT*, wildtype
